# *Calm with horses?* A systematic review of animal-assisted interventions for improving social functioning in children with autism

**DOI:** 10.1177/13623613221085338

**Published:** 2022-04-11

**Authors:** Jon H Sissons, Elise Blakemore, Hannah Shafi, Naomi Skotny, Donna M Lloyd

**Affiliations:** 1 University of Leeds, UK; 2 University of Manchester, UK

**Keywords:** animal-assisted interventions, autism, autism spectrum disorder, social functioning, systematic review

## Abstract

The aim of this systematic review was to evaluate the effect of animal-assisted interventions on social functioning in children with autism spectrum disorder, based on evidence from randomized control trials. Included studies were articles published in English, with school aged children from 4 to 18 years with autism spectrum disorder. Databases searched were MEDLINE, PsycINFO, EMBASE, Web of Science, CINAHL and Zoological Record. Data extraction from included studies included demographics and sample features, interventions and controls descriptions, outcome measures, study funding and descriptive statistics. Risk of bias was assessed, considering randomization, allocation concealment, blinding, attrition, selective reporting and other sources of bias. Studies were synthesized narratively based on the animal approach taken and the use of waitlist versus active controls. Nine studies were included reporting across eight trials. Studies overall reported improvements in social functioning following equine-assisted services, with preliminary evidence suggesting improvements are sustained in the short and medium term. Insufficient evidence was available to draw conclusions on the efficacy of other animal-assisted interventions. Future research should aim to address the limitations common to included designs.

**Lay abstract:**

Children with autism typically experience difficulties interacting socially with others when compared to their non-autistic peers. Establishing how effective interventions are for improving social functioning is important to help inform what should be offered to children with autism. This study reviewed how effective interventions that involved interaction with a live animal, known as animal-assisted interventions, are in improving social functioning in children with autism. A systematic search of the evidence on this topic found nine studies, which were explored for the effectiveness of animal-assisted interventions and the quality of methods used. Overall, these studies showed improvements in social functioning following equine-assisted or therapeutic horse-riding interventions, with initial evidence showing improvements are sustained in the short and medium term. However, several issues were identified, which limit the strength of any conclusions that can be drawn from this evidence. For example, in many studies people assessing the children were aware that they received the intervention or were in a control group. There was also not enough evidence available to draw conclusions on the effectiveness of other animal-assisted interventions. Future research should address the limitations that were common in the designs of these studies and investigate the potential benefit of other animal populations, such as dogs and cats.

Autism spectrum disorder (ASD) is a neurodevelopmental condition characterized by impairments in social interaction, communication and repetitive patterns of behaviour, interests or activities (American Psychiatric Association (APA), 2013). In the United Kingdom, 1.2% of 5 to 19 year olds were identified as having a diagnosis of autism (Marcheselli et al., 2018), with a higher proportion of parents (1.7%) reporting being told their child is on the autism spectrum by a health professional (Russell et al., 2014). Of those children receiving a diagnosis, there is a male to female ratio of 3:1 (Loomes et al., 2017). Children with autism also experience an increased likelihood of receiving other co-occurring diagnoses, most commonly attention deficit hyperactivity disorder, oppositional defiant disorder and anxiety (Brookman-Frazee et al., 2018; Simonoff et al., 2008). Rather than focussing on deficits and a diagnosis of ‘disorder’, many proponents within the autistic and research community favour a perspective of autism as reflective of neurodiversity (Baron-Cohen, 2017). Accordingly, calls to focus on improving quality of life and well-being in people with autism have been made in preference to treatments aiming to reduce autistic traits (den Houting, 2018). Nevertheless, for many children with autism, difficulties in interacting socially can present a range of immediate problems starting in education settings, such as experiences of exclusion (Pellicano et al., 2018) and bullying (Park et al., 2020).

A range of psychosocial interventions are currently recommended for use in children with ASD, aiming to increase joint attention, engagement and reciprocal communication (Lord et al., 2022; National Institute for Health and Care Excellence (NICE), 2013). However, existing interventions are not universally effective in children with autism (Jobin, 2020) and from the perspective of adults with autism, there is a greater willingness to take part in complementary interventions in the community over established socio-behavioural interventions such as Applied Behavioural Analysis (Benevides et al., 2020). One type of complementary intervention, acceptable to adults with autism and parents of children with autism (Benevides et al., 2020; London et al., 2020), is animal-assisted interventions (AAIs). AAIs incorporate the presence of a live animal, most frequently horses or dogs and, more rarely, other animals such as dolphins or guinea pigs (O’Haire et al., 2013) and are offered in many countries as complementary support for children with autism, including in the United States and United Kingdom (Eaton-Stull et al., 2020; Malcolm et al., 2018). AAIs are prominent in the public sphere, in media such as *The Horse Boy* (Isaacson, 2009) and *Calm with Horses* (Rowland, 2020). Proposed mechanisms of AAIs for children include reduction of stress – contact with animals has been shown to reduce anxiety in children (Crossman et al., 2020), and tactile contact may alter stress hormones, increasing peak oxytocin and reducing cortisol (Handlin et al., 2011). Reduced cortisol responses in children following AAIs may allow for reduced hyperactivity (Pan et al., 2019) and provide an ‘open’ context for children to engage with therapists and their environment (Malcom et al., 2018). Animals may also provide a less complex social stimulus for children with autism (Martin & Farnum, 2002) as their behaviour may be more predictable, and less challenging, as for example, animals can demand less eye contact than typical human interactions (Malcolm et al., 2018).

Despite their potential benefit, the evidence base for AAIs is limited (O’Haire, 2017). Previous systematic reviews have considered the impact of AAIs using less strict criteria, including lower quality evidence such as case study designs (O’Haire, 2017; O’Haire et al., 2013; Trzmiel et al., 2019), summarizing a range of preliminary and in some cases anecdotal evidence indicating AAIs may be beneficial for social functioning in children with ASD. In contrast, a meta-analysis including only higher quality randomized control trials (RCTs) had excluded AAIs from inclusion due to a limited number of trials and risk of bias concerns (Sandbank et al., 2019). To progress the evidence base for this potentially beneficial intervention, it remains important to evaluate the existing high-quality evidence. This systematic review addresses the gap in the literature by narratively synthesizing evidence on the effect of AAIs on social functioning in children with diagnoses of ASD based only on RCTs.

## Method

### Eligibility criteria

Studies eligible for inclusion were RCTs comparing AAIs to active controls without animal involvement or waitlist controls. Eligible studies included child participants of school age (from 4 to 18 years) with a diagnosis of ASD according to *Diagnostic and Statistical Manual of Mental Disorders* (5th ed.; DSM-5) criteria for ASD (APA, 2013). This included participants with prior diagnoses of Asperger’s or Pervasive Developmental Disorder Not Otherwise Specified (PDDNOS) as in the International Classification of Diseases (ICD-10; World Health Organization (WHO), 1993). Inclusion criteria required studies to report participant’s social behaviour as an outcome, assessed either by self-report or an external rater (parent, teacher, caregiver or other professional assessment) for both pre- and post- intervention. Dissertations and conference abstracts were excluded, as well as any studies without a live animal, such as virtual or robot animal interventions.

### Information sources

Searches were completed across six electronic databases on 28 October 2020; Ovid MEDLINE(R) (1946–present), APA PsycInfo (1806–present), Embase Classic+Embase (1947–present), Zoological Record (1978–2010), Web of Science (1900–present) and CINAHL(1960–present; via EBSCO databases). Search terms included variants of ‘Autism’ AND ‘Animal Intervention’ AND ‘Social Interaction’ AND ‘Child’ AND ‘Randomised control trial’, as shown in full in Appendix 1. When data were not available or more details about studies were needed, the corresponding author of each study was contacted, resulting in further data requests from Gabriels et al. (2015, 2018) and Souza-Santos et al. (2018). An updated search of the literature was performed covering five databases between 28 October 2020 and 8 October 2021; Ovid Medline (R) ALL 1946 to 8 October 2021, Embase Classic+Embase 1947 to 8 October 2021, APA PsycInfo 1806 to October 2021.

### Study selection

After removal of duplicates, remaining studies underwent abstract and title screening. Four researchers (E.B., J.H.S., N.S. and H.S.) each screened 252 abstracts, with any resulting disagreements discussed and resolved afterwards. Studies at this stage were removed if they had an adult sample, did not use live animals, included no ASD diagnosis, recorded no social outcome or were a previously missed duplicate. Remaining studies underwent full-text screening, with four researchers (E.B., J.H.S., N.S. and H.S.) each screening 15 or 20 articles with 10 articles overlapping (so that 36% of articles were double screened). Studies with quasi-experimental designs, lacking sufficient evidence of randomization and dissertations or conference abstracts were excluded at this stage.

### Data collection

Data extraction was completed using an adapted Cochrane Collaboration data extraction form. All nine included studies were double extracted and checked by two reviewers, with the first five checked by E.B. and J.H.S. and the latter four included studies by N.S. and H.S..

### Data items

From each study, the following information was extracted: sample demographics (including age, gender); sample features (verbal or non-verbal, diagnosis severity or description, intelligence quotient (IQ), prescribed medication); intervention and control description (components, staff involved in delivery, treatment timing, duration and frequency); outcome measures (relevant scale and subscales used, time points measured and reported, scale validity); study funding sources; and reported descriptive statistics with any associated *p* values. Where descriptive statistics were missing, authors were contacted via email requesting original data.

### Risk of bias in individual studies

To assess risk of bias in included studies, the Cochrane ‘Risk of Bias’ assessment tool was used by considering the criteria guidelines for each risk with respect to each study, or in the case of multiple outcomes per study, each outcome. Considered risks included selection bias, performance bias, detection bias, attrition bias and any other bias.

For selection bias, evidence of random sequence generation to avoid bias in the allocation to intervention and control groups was evaluated, as well as the concealment of these allocations to researchers so that they could not be predicted and influence procedure. For performance bias, the blinding of participants and personnel to the conditions participants were assigned to was considered, where interventions compared only to a waitlist control were assumed to be incompatible with blinding of participants. For detection bias, the blinding of outcome assessment was considered separately for each outcome measure where multiple were reported by a single study. For attrition bias, the incompleteness of reported outcome data was evaluated, indicated by a significant proportion of missingness or evidence of missingness related to the intervention or outcomes Missing Not At Random (MNAR). For reporting bias, reporting of results selectively was assessed, such as reporting based on significance or to support a hypothesis. Any other evident sources of bias were also considered, including baseline imbalances in measures or relevant characteristics, undeclared or inappropriate influence of study funding sources and specific sources of bias related to the design.

Risk of bias forms were completed for all included studies across each risk described, using ratings of low, high or unclear risk of bias.

### Synthesis of results

A narrative synthesis was used to bring together and summarize quantitative results across studies. Studies were described and analysed in terms of trial design, intervention content, outcome assessors, controls used and efficacy of results. No community involvement was incorporated into this process.

## Results

### Study selection

**Figure 1.**
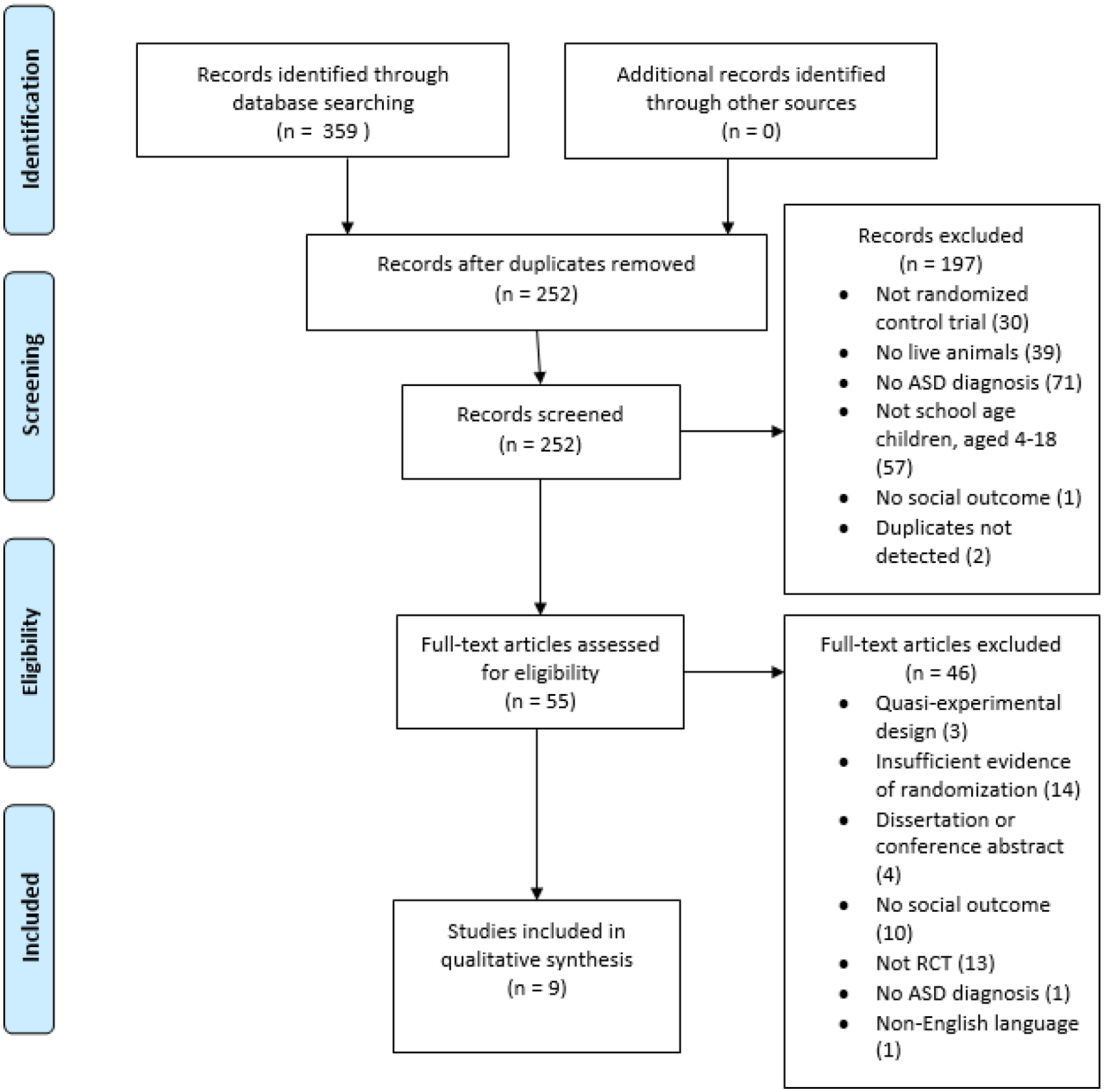
PRISMA flow diagram Source: Moher et al. (2009; www.prisma-statement.org).

Study selection produced nine studies eligible for inclusion involving eight trials. CINAHL, EMBASE, Medline, PsycInfo, Web of Science and Zoological Record databases were searched, producing a total of 359 studies. Two hundred and fifty-two studies remained after removal of duplicates. During abstract and title screening, 197 studies were removed for failing to meet inclusion criteria. Full texts for a total of 55 remaining studies were retrieved and screened, resulting in a further 46 studies excluded for failing to meet inclusion criteria. Complete inter-rater agreement was reached for articles, which were double screened. A final total of nine studies were selected for inclusion in narrative synthesis. Reasons for exclusion at each stage are detailed within the flow diagram of the study selection process (Figure 1) and listed individually in Appendix 2.

An updated search was performed from October 2020 to October 2021. The previous procedure was repeated, with 42 studies produced, 17 remaining after deduplication, of which seven were removed at title and abstract screening. Of 10 full texts screened, three further studies were selected for inclusion.

### Study characteristics

Of all nine included studies, eight reported unique RCTs, with one reporting a 6-month follow-up (Gabriels et al., 2018) to a previous trial (Gabriels et al., 2015). Seven out of the eight trials assessed the impact of an equine-assisted intervention, referred to as either therapeutic horse riding (THR; Bass et al., 2009; Gabriels et al., 2015; Pan et al., 2019) or equine-assisted therapy/activity (EAT/EAA; Borgi et al., 2016; Coman et al., 2018; Ozyurt et al., 2020; Souza-Santos et al., 2018). As recommended by Wood et al. (2021), the term equine-assisted services (EASs) will be used herein to describe different intervention approaches utilizing horses. One trial assessed the impact of a reading programme with the presence of dogs (Uccheddu et al., 2019). Although a higher proportion of non-equine-based AAIs were identified in a previous systematic review (O’Haire et al., 2013), many of these studies used single-subject or within participants designs and were, therefore, excluded from this review. In the trial using a dog-based intervention (Uccheddu et al., 2019), the lowest sample size of nine participants was reported, while sample sizes in the remaining equine-based studies ranged from 16 to 116. Three studies used *Diagnostic and Statistical Manual of Mental Disorders* (4th ed., text rev.; DSM-IV-TR; APA, 2000) criteria for ASD diagnosis (Bass et al., 2009; Borgi et al., 2016; Coman et al., 2018) and one used the more recent *DSM*-5 (APA, 2013; Uccheddu et al., 2019). Remaining studies used cut-off scores on the Autism Diagnostic Observation Schedule (ADOS/ADOS-2; Gabriels et al., 2015, 2018; Lord et al., 2012; Pan et al., 2019) or the Childhood Autism Rating Scale (CARS; Park & Kim, 2016; Souza-Santos et al., 2018), with the exception of the study by Ozyurt et al. (2020), which reported participant’s diagnosis of autism but not the diagnostic assessment used.

Although given different names, no differences between THR and EAT/EEA interventions were evident, and all EASs incorporated skills mounting and riding horses. EASs predominantly included a form of warm-up or preparation (Bass et al., 2009; Coman et al., 2018; Gabriels et al., 2015; Ozyurt et al., 2020; Pan et al., 2019; Souza-Santos et al., 2018) and skills caring for the horse (Borgi et al., 2016; Coman et al., 2018; Gabriels et al., 2015; Ozyurt et al., 2020; Pan et al., 2019). Some studies included additional components, such as mounted games (Bass et al., 2009), drawing activities (Pan et al., 2019) or specific time for ‘touch stimulation’ (Souza-Santos et al., 2018).

Equine-based studies predominantly used a waitlist control group, with the exceptions of a barn activity (BA) control without horse interaction in two studies (Gabriels et al., 2015; Pan et al., 2019) and a dance group control within a crossover design in one study (Souza-Santos et al., 2018). In the study by Uccheddu et al. (2019), dog-assisted reading was compared to reading in the absence of a dog. Full study characteristics are reported in Table 1.

**Table 1.** Summary characteristics of studies included in review (*n* = 9).

Author(s)	Study design	Sample demographics	Sample features	Intervention	Intervention components	Duration	Control	Outcome measure	Effect of intervention
Bass et al. (2009)	RCT	*N* = 36 Male (29) Female (5) Mean age in years (6.89)	Verbal (41.7%) Nonverbal (19) Diagnosis = Asperger’s (2) Mild (11) Moderate (16) Severe (5) No IQ, co-occurring diagnoses or medication information provided	Therapeutic horse riding	Mounting/dismounting Warm up exercises Riding Skills Mounted Games	12 weeks	Waitlist control	Social Responsiveness Scale (SRS)	Effect of improved social motivation
Borgi et al. (2016)	RCT	*N* = 28 Male (28) Female (0) Age (*M, SD*): Experimental (9.2, 1.8), Control (8.0, 1.5)	Verbal (100%) IQ (*M, SD*): Intervention (98.3, 16.2) IQ control (92.8, 19.9) No severity, co-occurring diagnoses or medication information provided	Equine- assisted therapy	Grooming/hand walking Riding skills Ground/closure phase	25 weeks	Waitlist control	Vineland Adaptive Behaviour Scale (VABS)	Effect of improved social functioning
Coman et al. (2018)	RCT	*N* = 50 Male (42) Female (8) Age (*M, SD*): Experimental (8.84, 1.72) Control (8.56, 1.50)	Verbal (38). No IQ, severity, co-occurring diagnoses, or medication information provided	Equine- assisted activities	Warm up Riding skills Individual and group games on the horse Grooming activities	12 weeks	Waitlist control	Social Responsiveness Scale (SRS)	Effect of improved social functioning and some maintenance at 8-week follow-up
Gabriels et al. (2015)	RCT	*N* = 116 Male (101) Female (15) Age (*M, SD)* Experimental (10.5, 3.2), Control (10.0, 2.7)	non-verbal IQ (NVIQ; *M, SD*): Intervention (86.7, 25.5) Control (86.1, 22.7) Community psychiatric diagnosis: Intervention (48.3%) Control (48.3%). No severity, verbal ability or specific medication information provided	Therapeutic horse riding	Warm up Therapeutic riding skills (mounting, halting, steering, running, trotting) Horsemanship skills (how to lead and care for horse) Cool down	10 weeks	Barnyard Activity Control	Social Responsiveness Scale (SRS)	Effect of improved social cognition and social communication in intervention group compared to barnyard activity control
Gabriels et al. (2018)	RCT 6-month follow-up	*N* = 64 Male (54) Female (10) Age (*M, SD*): Experimental (10.7, 2.9) Control (9.4, 2.5)	NVIQ (*M, SD*): Intervention (88.4, 25.1) Control (89.2, 19.8) Community psychiatric diagnosis: Intervention (50%) Control (39%). No severity, verbal ability or specific medication information provided	Therapeutic horse riding	Warm up Therapeutic riding skills (mounting, halting, steering, running, trotting) Horsemanship skills (how to lead and care for horse) Cool down	10 weeks	Barnyard Activity Control	Social Responsiveness Scale (SRS)	Effect of improved social cognition and social communication persisted after 6 months in intervention group
Hernández-Espeso et al. (2021)	RCT	*N* = 43 Male (33) Female (10)	None had acquired brain injuries, frequent seizures or a diagnosis of ‘Asperger’s Syndrome’. No severity, co-occurring diagnoses, IQ or medication information provided	Dolphin-assisted therapy	Preparation Safety reminder Open play Feeding Dolphin activities	6 weeks	Therapy without dolphin	Vineland Adaptive Behaviour Scale 2 (VABS-II)	No significant improvements in comparison to active control
Ozyurt et al. (2020)	RCT	*N* = 24 Male (17) Female (7) Age: 4–12 years, *M* *=* 6.77	None had genetic syndromes, epilepsy or mild or moderate intellectual disability. No psychotropic drugs taken. No severity or verbal ability information	Equine-assisted activities	Preparation Warm-up Mounting Main session Finishing	8 weeks	Waitlist control	Social Communication Questionnaire (SCQ)	Positive effect of intervention for the experimental group. No effect for control group
Pan et al. (2019)	RCT	*N* = 16 Male (13) Female (3) Age (*M, SD*): Experimental (11.88, 2.45) Control (9.80, 2.82)	IQ (*M, SD*): Intervention (102.88, 16.28) IQ control (100.25, 29.26) Community psychiatric diagnosis: Intervention (100%) Control (50%). Psychotropic medication: Intervention (75%) Control (37.5%) All children non-verbal IQ (NVIQ) ⩾ 40. No severity information	Therapeutic horse riding	Sit with a volunteer Start group Review group schedule Warm up exercises Lesson and activity Cool down exercises Therapeutic horse riding (THR) group dismount and thank horses All groups thank volunteers Drawing activity at table	10 weeks	No horse interaction barn activity control	Systematic Analysis of Language Transcripts (SALT) Social Responsiveness Scale (SRS) Aberrant Behavior Checklist–Community (ABC-C)	Positive effect on social awareness and social communication behaviours for the experimental group compared to the control group
Peters et al. (2021)	RCT	*N* = 24 Male (16) Female (5) Age: 6–13 years	ABAS-GAC (Mean, *SD*) Waitlist (68.44, 10.00), Horseplay (73.92, 9.17) ADHD (9) Anxiety (5) OCD (1)	Occupational therapy in an equine environment (OTee)	Greetings Activities with horses Goodbyes Caregiver debrief	10 weeks	Waitlist Occupational Therapy in a Garden Environment (OTGE)	Social Responsiveness Scale 2 (SRS-2)	Effect of improved social motivation in comparison to control
Souza-Santos et al. (2018).	RCT	*N* = 45 Male (36) Female (9) Age: *M* *=* 7.09	More than 31 points on CARS scale. Taking additional medication: Risperidone 1 mg per mL oral solution per day (88.8%) Carbamazepine 500 mg/tablet per day (13.3%) Pericyazine 20 mg/tablet per day (6.6%) No IQ, co-occurring diagnoses or verbal ability information	Equine- assisted therapy	Horse approach Touch stimulation ride and course with varied riding. Rode a horse with verbal commands and visual clues	12 weeks	Dance group control and equine and dance control	Child autism rating scale (CARS), Functional Independence Measure (FIM), WHO disability Assessment Scale (Social participation)	Positive effects on autism degree, functionality and social participation for the experimental group
Uccheddu et al. (2019).	RCT	*N* = 9 Male (7) Female (2) Age: *M* *=* 7.60	Severity of ASD: Intervention (4 require support, 1 require substantial support) Control (3 require support, 1 require substantial support). IQ (*M, SD*): Intervention (75.2, 16.4) Control (108.2, 24.7). No co-occurring diagnoses, verbal ability or medication information	Reading programme with the presence of dogs	Read a book one-to-one with a dog. Child–animal interaction was limited to only verbal contact	70 days	Reading without a dog control	Vineland Adaptive Behaviour scale	No improvements in social skills in both groups. Children’s engagement in social interactions did not increase
Zhao et al. (2021)	RCT	*N* = 61 Male (44) Female (17) Age: 6 – 12 years. Age: *M* = 7.10	No IQ, co-occurring diagnoses, severity, verbal ability or medication information provided	Therapeutic horse riding	Warm up Riding skills and horsemanship instruction Therapeutic horse-riding exercises and activities Cool down and reward	16 weeks	Routine activities as usual	Social Skills Improvement Rating Scales (SSIS-RS)	Effect of improved social skills score in intervention group compared to control

RCT: randomized controlled trial; IQ: intelligent quotient; ABAS: Adaptive Behaviour Assessment Scale; GAC: general adaptive composite; ADHD: attention deficit hyperactivity disorder; OCD: obsessive compulsive disorder; ASD: autism spectrum disorder.

### Risk of bias within studies

**Figure 2.**
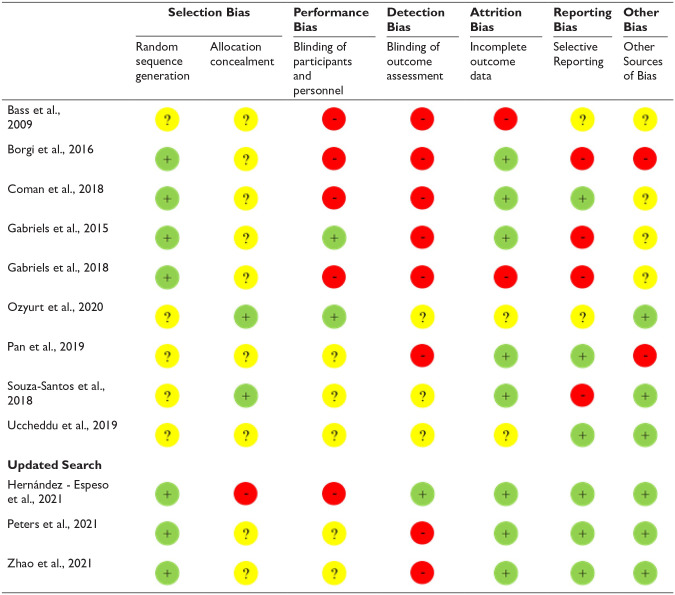
Risk of bias judgements for each study. Green circle (+) = low risk, red circle (−) = high risk, yellow circle (?) = unclear risk.

Included studies were assessed using the Cochrane Risk of Bias tool and assigned either ‘low’, ‘high’ or ‘unclear’ risk of bias for each risk. Unclear risk of bias was assigned where studies did not describe sufficient details, such as the randomization method, allocation concealment or blinding. Only one study was judged to not have any high risk of bias, although risk of bias was unclear for four of the risks for this study. All remaining studies had a mixture of low, high and unclear risks of bias. Detection bias was a consistent issue across studies, with no studies at low risk of bias for adequately blinding outcome assessment, often due to assessment by parents or teachers inevitably aware of group assignment. Judgements for risk of bias across each risk across all nine studies are shown in Figure 2.

### Synthesis of results

#### Efficacy within equine-based approaches

Seven of eight original studies evaluated EASs, with four of these assessing social outcomes with the Social Responsiveness Scale (SRS; Constantino et al., 2003). All of these reported significant improvements in SRS total scores, but results varied across different SRS subscales, with significant improvements in social motivation (Bass et al., 2009; Coman et al., 2018), social communication (Coman et al., 2018; Gabriels et al., 2015; Pan et al., 2019), social cognition (Coman et al., 2018; Gabriels et al., 2015), social awareness (Pan et al., 2019) and autistic mannerisms (Coman et al., 2018) all reported. Coman et al. (2018) reported significant improvement on four of five subscales of the SRS in a sample of 50. However, by contrast, the largest powered study of 116 participants by Gabriels et al. (2015) only reported significant improvements in social cognition and social communication subscales. Pan et al. (2019) aimed to replicate the intervention previously evaluated in the study by Gabriels et al. (2015); however, the subscales of the SRS, which significantly improved were inconsistent between these studies. There is, therefore, limited evidence to suggest that variance in subscale improvement was related to heterogeneity in intervention delivery. The remaining three studies reported significant improvements in the Vineland Adaptive Behaviour Scale (VABS; Sparrow et al., 1984) socialization subscale (Borgi et al., 2016), social participation (Souza-Santos et al., 2018) and Social Communication Questionnaire (Avcil et al., 2015) communication subscale (Ozyurt et al., 2020). Although as previously described, there was some variation in the content of EAS, with some interventions including additional activities (Bass et al., 2009), the mechanisms proposed to be beneficial within the literature (such as tactile contact with animals, relaxation with animals and skills learning) were incorporated into all approaches through riding and horsemanship activities with horses.

Although Gabriels et al. (2018) reported on a 6-month follow-up to a previous trial (Gabriels et al., 2015), as SRS descriptive statistics were not reported for follow-up, the authors were contacted requesting data. Results showed that across SRS subscales, which significantly improved in the study by Gabriels et al. (2015), SRS communication and SRS cognition remained over twice the standard error below mean scores post treatment, while social awareness scores increased above post-treatment mean (Gabriels & Pan, Personal communication, 7 December 2020). Coman et al. (2018) also collected follow-up 8 weeks post intervention, retaining 50% (25/50) of the sample and reporting sustained improvements in SRS total, social cognition, social communication and autistic mannerisms.

In an updated search from 2020 to 2021, two further studies evaluating equine-based approaches were identified. Zhao et al. (2021) reported improvements in Social Skills Improvement System Rating Scales (SSIS-RS) assessed social skills in comparison to a routine activity control in 61 children receiving a 16 week protocol of THR. Peters et al. (2021) evaluated an Occupational Therapy within an equine environment in comparison to a waitlist control involving Occupational Therapy in a garden environment. Consistent with some studies (Bass et al., 2009; Coman et al., 2018), they reported improvements in social motivation, but not other domains of the SRS.

#### Efficacy in non-equine-based approaches

As only one intervention assessed the impact of a dog-based intervention, comparisons cannot be drawn between intervention components. In this intervention, Uccheddu et al. (2019) randomized nine children to either a reading with dogs group or reading without dogs group, where children were instructed to read the same book on a weekly basis. Physical contact with the dogs was not allowed; potential mechanisms of change instead involved reading and talking to the dogs, which was suggested to be beneficial by providing a non-judgmental environment to practice reading in, with emotional support from the dogs actively listening. Sessions were conducted in the presence of a psychologist; otherwise, the intervention included no other targeted mechanisms or skills. Two female dogs were selected for their suitability for the intervention, based on their cooperation with children, reduced anxiety and aggression. The intervention partly aimed to improve reading abilities; however, in terms of social outcome, no significant improvements on the VABS socialization were reported in the reading with dogs’ group (Uccheddu et al., 2019). Notably, this intervention focussed on improving reading skills with social communication as a secondary outcome, whereas previous interventions used in case studies delivered dog-assisted interventions programmes focused on social skills (Silva et al., 2011). Results across all animal approaches are reported in full in Table 2.

**Table 2.** Study results.

Author(s)	Intervention	Control type	Duration	Outcome measures	Results	
					Intervention (*M, SD*)	Control (*M, SD*)
Bass et al. (2009)	Therapeutic horse riding	Waitlist	12 weeks	Social Responsiveness Scale (SRS) Total Subscales: Social Cognition Social Awareness Social Motivation	Pre (85.9, 37.5) Post (73.6, 24.1) *p* = **0.017** Pre (20.8, 7.3) Post (16.1, 5.8) Pre (12.1, 4.7) Post (9.9, 2.7) Pre (17.3, 7.1) Post (12.5, 5.9)	Pre (89.3, 35.4) Post (94.4, 32.1) *p* = 0.916 Pre (11.5, 3.6) Post (18.9, 6.6) Pre (11.5, 3.6) Post (11.1, 3.2) Pre (18.2, 7.1) Post (16.2, 6.7)
Borgi et al. (2016)	Equine-assisted therapy	Waitlist	25 weeks	Vineland Adaptive Behaviour Scale (VABS) Socialization	Change Post–Pre (0.72, 0.22) *p* = 0.034^a^	Change Post–Pre (0.23, 0.21)
Coman et al. (2018)	Equine-assisted activities	Waitlist	12 weeks	Social Responsiveness Scale (SRS) Teacher Reported Total Subscales: Social Cognition Social Awareness Social Motivation Social Communication Autistic Mannerisms	Pre (99.4, 25.3) Post (74.0, 25.8) *p* < **0.001**, *d* = 1.23 Follow-up (78, 27.4) Pre (19.0, 5.1) Post (15.4, 5.5) *p* < **0.001** *d* = 0.82 Follow-up (15.7, 6.0) Pre (11.7, 2.7) Post (9.8, 2.8) *p* = 0.153 Follow-up (9.6, 3.2) Pre (16.1, 6.2) Post (11.2, 5.1) *p* < **0.001** *d* = 0.97 Follow-up (11.7, 6.0) Pre (33.6, 9.6) Post (24.1, 10.2) *p* < **0.001** *d* = 1.26 Follow-up (26.6, 10.1) Pre (17.5, 7.7) Post (11.7, 5.0) *p* < **0.001** *d* = 0.92 Follow-up (14.5, 5.9)	Pre (93.9, 35.0) Post (101.0, 31.0) *p* = 0.13 Follow-up (88.4, 37.0) Pre (18.1, 6.6) Post (19.5, 6.1) Follow-up (18.3, 7.3) Pre (11.6, 4.7) Post (12.4, 4.3) Follow-up (9.9,4.2) Pre (15.9, 7.9) Post (16.0, 6.9) Follow-up (13.5, 8.1) Pre (31.8, 13.0) Post (34.7, 12.0) Follow-up (28.8, 13.0) Pre (16.3, 8.2) Post (17.7, 7.5) Follow-up (17.7,7.9)
Gabriels et al. (2015)	Therapeutic horse riding	Barnyard activity	10 weeks	Social Responsiveness Scale (SRS) Subscales: Social Cognition Social Awareness Social Motivation Social Communication Autistic Mannerisms	Pre (20.3, 5.63) Post (17.6, 5.55) *p* = **0.003**^a^ Pre (13.7, 3.16) Post (12.2, 3.14) *p* = 0.054^a^ Pre (15.8, 5.88) Post (11.9, 4.97) *p* = 0.19^a^ Pre (36.8, 10.04) Post (30.2, 8.75) *p* = **0.003**^a^ Pre (21.2, 6.36) Post (18.4, 6.04) *p* = 0.61^a^	Pre (19.3, 5.58) Post (19.1, 5.64) Pre (13.2, 3.54) Post (12.4, 3.36) Pre (15.2, 5.09) Post (13.2, 6.36) Pre (33.9, 8.84) Post (3.36, 1.38) Pre (21.2, 6.30) Post (19.4, 6.37)
Gabriels et al. (2018)	Therapeutic horse riding	Barnyard activity	10 weeks	Social Responsiveness Scale (SRS) Subscales: Social Cognition Social Awareness Social Motivation Social Communication Autistic Mannerisms	Pre (19.7, 5.51) Post (17.1,5.41) Follow-up (16.4, 6.15) Pre (13.5,3.28) Post (11.6,3.22) Follow-up (12.0, 3.86) Pre (15.0, 5.24) Post (12.1, 4.89) Follow-up (12.4, 5.57) Pre (36.1, 9.14) Post (29.3, 7.72) Follow-up (28.4, 11.85) Pre (20.5, 5.16) Post (18.1, 4.65) Follow-up (17.0, 6.24)	
Hernández-Espeso et al. (2021)	Dolphin-assisted therapy	Therapy without dolphins	6 weeks	Vineland Adaptive Behaviour Scale 2 Socialization Communication	Pre (64.83, 16.27) Post (70.21, 16.07) Pre (76.88, 25.99) Post (80.42, 25.87)	Pre (70.11, 12.93) Post (73.74, 16.06) Pre (78.05, 25.87) Post (81.05, 29.9)
Ozyurt et al. (2020)	Equine-assisted activities	Waitlist	8 weeks	Social Communication Questionnaire (SCQ), cut-off > 15 requires full ASD screening	Pre (19.92, 4.12) Post (18.25, 3.70) *p* = **0.0003**	
Pan et al. (2019)	Therapeutic horse riding	No horse interaction barn activity	10 weeks	Social Responsiveness Scale (SRS)Subscales: Social awareness Social cognition Social communication Autistic mannerisms Social Motivation	Pre (15.43, 3.95) Post (11.29, 1.38) *p* = **0.01**^a^ ES^b^ = − 1.74 Pre (20.43, 7.11) Post (21.29, 3.30) *p* = 0.72^a^ ES = − 0.22 Pre (41.00, 9.33) Post (34.57, 3.95) *p* = **0.03**^a^ ES = − 1.46 Pre (21.71, 6.05) Post (20.29, 4.96) *p* = 0.35^a^ ES = − 0.57 Pre (18.57, 3.87) Post (16.43, 4.28) *p* = 0.18^a^ ES = − 0.83	Pre (12.29, 2.56) Post (13.57, 4.12) Pre (16.86, 6.87) Post (18.71, 7.43) Pre (29.29, 9.83) Post (31.29, 10.98) Pre (17.29, 5.12) Post (18.86, 6.47) Pre (12.71, 5.96) Post (12.71, 6.05)
Peters et al. (2021)	Occupational Therapy in an Equine Environment (OTee)	Waitlist Occupational Therapy in a Garden Environment (OTGE)	10 weeks	Social Responsiveness Scale 2 Social awareness Social cognition Social communication Social motivation	Pre (69.45, 10.39) Post (68.9, 8.03) *p* = 0.78 *d* = − 0.006 Pre (72.10, 8.04) Post (72.30, 9.24)	Pre (76.89, 10.90) Post (74.67, 10.72) *p* = 0.38 *d* = − 0.31 Pre (77.56, 7.45) Post (76.56, 6.86)
					*p* = 0.914 *d* = − 0.02 Pre (73.7, 8.51) Post (71.1, 7.15) *p* = 0.096 *d* = − 0.39 Pre (69.85, 9.39) Post (66.75, 12.39) *p* = **0.033** *d* = − 0.51	*p* = 0.69 *d* = − 0.14 Pre (78.67,4.85) Post (78.22, 7.50) *p* = 0.88 *d* = − 0.06 Pre (74,67, 8.20) Post (71.00, 7.86)
Souza-Santos et al. (2018).	Equine-assisted therapy (EAT)	Dance group (D) and Equine and dance group (EAT&D)	12 weeks	WHO disability Assessment Scale (Social participation)	EAT = Pre (2.25, 0.13) Post (1.88, 0.3) *p* *=* **0.03**	D = Pre (2.51, 0.25) Post (1.83, 0.52) *p* *=* **0.04** EAT&D = Pre (2.63, 0.15) Post (1.03, 0.08) *p* < **0.0001**
Uccheddu et al. (2019).	Reading programme with the presence of dogs	Reading without a dog	70 days	Vineland Adaptive Behaviour subscales (VABS): Total Communication Daily Living skills Socialization Motor skills	Pre (57.3, 19.6) Post (76.3, 29.2) *p* > 0.05 Pre (69.2, 25.8) Post (97.0, 36.7) Pre (45.0, 8.3) Post (76.3, 29.6) Pre (50.0, 17.1) Post (62.6, 22.1) Pre (46.5, 9.1) Post (48.0, 0.0)	Pre (63.4, 26.1) Post (78.5, 34.6) *p* > 0.05 Pre (74.8, 29.8) Post (99.0, 45.2) Pre (50.4, 10.7) Post (78.0, 36.8) Pre (55.0, 19.0) Post (65.5, 21.9) Pre (40.0, 0.0) Post (55.0, 0.0)
Zhao et al. (2021)	Therapeutic horse riding	‘Routine activities’	16 weeks	Social Skills Improvement System Rating Scales (SSIS-RS) Total	Pre (44.68, 7.48) Post (50.87, 6.47) *p* < **0.001**^a^ **ES** = **0.421**	Pre (44.27, 4.31) Post (45.43, 5.08)

a *p*-values reported for time × group interaction.

b Effect size calculated (2 × t-value)/√df from the contrast of the time × group interaction.

*p* < 0.05 are indicated in bold.

An updated search also identified another study taking a non-equine-based approach by Hernández-Espeso et al. (2021) in which dolphin-assisted therapy (DAT) was delivered to 48 children with ASD, involving structured games and activities in water equivalent to those with horses in equine-assisted services. Significant improvements in VABS 2 socialization were reported in the DAT group; however, these improvements were not significantly different to those found in an active therapy without dolphins control.

#### Efficacy in studies using active versus waitlist controls

Of eight included studies, four utilized waitlist controls (Bass et al., 2009; Borgi et al., 2016; Coman et al., 2018; Ozyurt et al., 2020) and four used active controls (Gabriels et al., 2015; Pan et al., 2019; Souza-Santos et al., 2018; Uccheddu et al., 2019). Bass et al. (2009) delivered a 12-week EAS programme to 36 children, diagnosed with mild-to-severe ASD and Asperger’s, resulting in improved social motivation on the SRS. Coman et al. (2018) also delivered an EAS intervention for a period of 12 weeks in a sample of 50, predominantly male children with autism. Again, they reported improvements in social functioning on the SRS, with some sustained changes in SRS total, social cognition, social communication and autistic mannerisms at 8-weeks follow-up. Borgi et al. (2016) delivered EAS to 28 boys over 25 weeks, reporting improved social functioning on the VABS. All three of these studies were limited by high risk of performance bias, as blinding was not possible due to use of waitlist controls.

Ozyurt et al. (2020) successfully blinded personnel but not participants; however, in this context, children are not expected to have expectations of intervention effects and are, therefore, of less concern as a source of risk of bias. Gabriels et al. (2018) reported the effects of a 10-week EAS in the largest sample of 116 children, in comparison to a barnyard activity control. Results demonstrated significant improvements in social functioning measured by SRS total score, as well as social cognition, social communication and social awareness subscales, which were sustained at a 6-month follow-up in 64 of these participants in social cognition and social communication (Gabriels et al., 2015, 2018). As Pan et al. (2019) replicated this procedure in a smaller sample of 16 children aged 6–16 years, they utilized the same control, where participants interacted with a life-sized stuffed horse in a barn to learn horsemanship skills without any live horse interaction. Pan et al. (2019) reported improvements in social functioning, but in this case only in SRS total, SRS awareness and SRS communication. Souza-Santos et al. (2018) utilized a crossover design, in contrast to the parallel designs used in all other included studies. In this study, the efficacy of an EAS was evaluated in comparison to a dance group control, as well as a combined equine and dance control over a 12-week period delivered to 45 children. Results demonstrated improved social participation as measured by the WHO Disability Assessment Scale (Huang et al., 2017) after receiving EASs in comparison to the dance group control.

Finally, Uccheddu et al. (2019) evaluated the impact of a non-equine-based approach, comparing the impact of a dog-assisted reading programme to a programme of reading without a dog over 10 weeks, in a sample of nine children. Results from this study demonstrated non-significant improvement in social skills in either group on the VABS. Although this meant that three out of four studies using active controls reported significant effects in comparison to four out of four studies using waitlist controls, it is difficult to draw any conclusions on this basis as the latter study was the only one to not evaluate an equine-based intervention. Studies using active controls nevertheless reduced the chance of reporting overinflated outcome effects, by controlling for the possibility of benefits to social functioning by engaging in activities within an intervention rather than remaining on a waitlist. Risk of performance bias was low in some of these studies using active controls (Gabriels et al., 2015), as blinding of participants and personnel was more feasible as a result of using active controls.

#### Efficacy in studies using parent, teacher or caregiver versus clinician reports

Of the included studies, the majority collected outcomes using either parent (Bass et al., 2009; Borgi et al., 2016), caregiver (Gabriels et al., 2015, 2018) or teacher assessment (Coman et al., 2018). The assessors collecting outcomes were unclear in Souza-Santos et al. (2018) as well as Ozyurt et al. (2020) who may have used a clinician assessor. Only one study unambiguously reported use of a clinician evaluator (Uccheddu et al., 2019).

As Coman et al. (2018) collected both parent and teacher report, only teacher report was extracted, assuming parents may be less impartial and more susceptible to bias than teacher reports (Jones et al., 2017). Nevertheless, Coman reported statistically significant (*p* < 0.05) reliability coefficients between parent and teacher reports for each aspect of the SRS, except for the autistic mannerism’s subscale. Despite this agreement between raters, of more concern is the extremely limited number of studies using independent evaluators to assess outcomes. As the one clear exception also reported no significant improvements in social outcomes (Uccheddu et al., 2019), there is limited evidence to exclude the possibility that reported results are influenced by bias in outcome assessors. However, as this study was also the only study to evaluate a dog-assisted intervention, no comparisons based on outcome assessors can be made between EASs.

#### Efficacy in studies with low risk of bias

None of the included studies were at low risk of bias consistently across all risk of bias judgements. Although two studies (Ozyurt et al., 2020; Uccheddu et al., 2019) received no high risk of bias judgements, the number of unclear risks for these studies renders any focus on these studies inappropriate, as risk of bias that is less apparent is not necessarily any less likely to be high.

## Discussion

### Summary of evidence

Overall, across a small number of studies, this systematic review found some evidence of the efficacy of EASs in improving social functioning in children with autism, but insufficient evidence of the benefits of AAIs more broadly. Most included studies evaluated the efficacy of EASs, with all reporting significant improvements across varied measures of social functioning, but some inconsistencies in changes in subscales of the SRS across those reporting this outcome. In two studies reporting follow-up outcomes, improvements in social communication and social cognitions remained significant at 8 weeks and 6 months post intervention. Included interventions were similar to those in earlier reviews; between 8 and 12 weeks in duration and involving an approximate average of 10 h contact for participants (O’Haire, 2017). All nine primary studies within the present review utilized RCT designs; however, multiple study limitations were prevalent – risks of bias were identified, namely that 66% of studies were at high risk of detection bias and 44% of studies were at high risk of performance and reporting bias. Given these limitations, caution should remain in drawing strong conclusions from this evidence and further trials should aim to minimize these sources of bias.

Included studies also provided limited evidence for any mechanisms of change underlying a beneficial effect of AAIs on social functioning. One proposed mechanism of change is that AAIs function as calming stimuli reducing stress responses (O’Haire, 2017), which can be a source of difficulty in social interactions in children with autism (Corbett et al., 2010). Pan et al. (2019) measured salivary cortisol before and after children received EAS or a barnyard activity control. Although changes in post-session cortisol over the 10-week period did not occur, there were significant pre- to post-session reductions in cortisol in the EAS group. These changes were associated with improvements in irritability and hyperactivity, although no equivalent analysis was performed for social outcomes. While this provides some evidence of the role of AAIs in reducing stress hormones, whether this is associated with an improved ability to develop social skills remains uncertain.

In the one included study evaluating the impact of a dog-assisted intervention, no tactile contact was allowed between children and the dogs, which may have removed the benefit of stress reduction in AAIs (Handlin et al., 2011). This was the only included study, which reported no significant improvements in children’s social functioning following the intervention (Uccheddu et al., 2019); however, there should be caution in comparing dog- and equine-assisted interventions and further evidence is required to draw conclusions on the efficacy of dog-assisted approaches. Rather than acting primarily as a reading programme (Uccheddu et al., 2019), other dog-assisted interventions within the literature instead aim to improve social skills in children with autism and allow tactile contact as a possible beneficial mechanism (Silva et al., 2011) and, therefore, might produce a different effect.

An update to the literature search produced three further studies, two of which provided results consistent with previous trials demonstrating improvements in socialization in children with ASD receiving equine-assisted services (Peters et al., 2021; Zhao et al., 2021). These studies were, however, limited by similar issues identified in previous trials, such as a lack of blinding in outcome assessment. The remaining study by Hernández-Espeso et al. (2021) evaluated the efficacy of a dolphin-assisted intervention and reported significant improvements, which did not differ significantly from an active control. This demonstrates the importance of trials using active controls for animal-assisted interventions, especially in the case of ‘exotic’ animal interventions where costs are likely to be significantly higher than equivalent interventions without animals.

### Limitations

Despite our focus on RCTs, improvements to the rigour of research methods used could still be made, such as clearer reporting of randomization methods used. While random allocation to groups is preferable to non-randomized designs, many included studies used waitlist rather than active controls as comparison groups (Bass et al., 2009; Borgi et al., 2016; Coman et al., 2018; Ozyurt et al., 2020). Waitlist controls may inflate reported effect sizes (Michopoulos et al., 2021) and active controls may provide an opportunity to reduce risk of bias by better enabling blinding of participants to their group allocation. High risks of bias were a persistent issue across most studies, with consistent issues with detection bias. Many studies failed to adequately blind outcome assessment, largely due to the use of parent- or carer-recorded outcome measures, which is a notable limitation within the literature on autism interventions for children (Jones et al., 2017). Efforts to provide blinded assessment of outcomes in RCTs are arguably the most essential design improvement for future RCTs to make in this area. As no restrictions on sample size were included, some studies may also have been underpowered to detect any significant effects, such as a sample of only nine children in the study by Uccheddu et al. (2019). In terms of the review itself, as it was not preregistered, this introduces the potential for bias resulting from any changes made to the method. All procedures were kept the same throughout the trial with the exception of GRADE ratings for the overall body of evidence, which were removed from the discussion.

There are also a series of practical limits to the results reported across included studies. Scaling up EASs could present practical challenges, as for example, in the largest scale study, Gabriels et al. (2015) delivered an EAS in sessions of two to four participants at a time. As the intervention required trained staff, volunteers and animals, the resource constraints of a riding centre could limit the expansion of EAS to larger scales. In the study by Pan et al. (2019), children with uncontrolled seizures were unable to participate due to risk of danger during horse-riding. As there are higher rates of epilepsy in people with autism than the general population (Spence & Schneider, 2009), risk of seizures may exclude a significant portion of children with autism from participation in AAIs. Generalizability of AAIs is also limited, as subgroups of children with autism were excluded from many studies, such as children with intellectual disability (Borgi et al., 2016; Gabriels et al., 2015, 2018; Ozyurt et al., 2020; Pan et al., 2019). Of the remaining studies, only Uccheddu et al. (2019) reported the mean IQ of the sample. While some studies included only verbal children with autism (Borgi et al., 2016), improvements in social functioning in mixed samples of both verbal and non-verbal children with autism have been demonstrated (Bass et al., 2009; Coman et al., 2018).

Although the present review was limited to a narrative synthesis and not a meta-analysis, it acts as a stop gap in evaluating the efficacy of AAIs for social functioning in children with autism as the quality of available evidence improves. In subsequent years, further RCTs, which build upon the limitations highlighted in the present review ought to be reviewed and synthesized in a meta-analysis to estimate the size of effects on social communication and provide guidance for the most effective intervention.

## Conclusion

This review reported on evidence from nine RCTs, many of which were published in recent years and have not been included in previous systematic reviews (O’Haire et al., 2013, 2017; Trzmiel et al., 2019). We found evidence to support the efficacy of the most prominent form of AAI – EASs – in improving social functioning in children with autism. A small amount of evidence supported the continuation of benefits in social functioning at short- (8-week) and medium-term (6-month) follow-ups. Insufficient evidence was available to conclude on the efficacy of other AAIs such as those including dogs. Similarly, no comparisons could be made between outcomes based on the measures used. Future studies should aim to address the limitations common to included designs.

## Appendix 1

Search terms

ASD or Autis* or Pervasive development disorder or PDD or PDDNOS or Learning disabilit* or Neurodevelopmental disorder or Asperger*

AND

Animal assisted or Animal intervention or Animal therapy or Animal assisted or Animal facilitated or Anthrozoology or Assistance animal* or Assistance dog* or Assistance horse* or Canine therapy* or Canine assisted or Canine facilitated or Companion animal* or Dog therapy or Dog assisted or Dog facilitated or Dolphin therapy or Dolphin assisted or Dolphin facilitated or Equine therapy or Equine assisted or Equine facilitated or Hippotherapy or Horseback riding or Human animal bond or Human animal interaction* or Pet therapy or Pet assisted or Pet facilitated or Service animal* or Service dog* or Service horse* or Therapeutic animal* or Therapeutic dog* or Therapeutic horse* or Therapeutic horseback or Therapeutic pet* or Therapeutic riding

AND

Children or Child or Child development or Adolescen* or Young people or Girl* or Boy* or Youth

AND

Assign* or Control group or BAU or Wait list or RCT or Random* or quasi or Treatment group or Intervention group or Group design or trial

## Appendix 2

Full-text exclusion list

55 Full texts, search – 28 October 2020.

**Table:**

Reference	Reason for exclusion
Adalarasu, K., Jagannath, M., & James, O. (2020). Assessment of techniques for teaching school children with autism. *IRBM, 41*(2), 88–93.	Quasi-experimental design
Albasha, H., Kelly, M., Andrews, J., & Rice, S. (2016). The effects of animal assisted intervention on the social initiation behaviors of children with an autism spectrum disorder. *Journal of Investigative Medicine, 64*(1), 264.	Insufficient evidence of randomization
Anderson, S., & Meints, K. (2016). Brief Report: The effects of equine-assisted activities on the social functioning in children and adolescents with autism spectrum disorder. *Journal of Autism and Developmental Disorders, 46*(10), 3344–3352.	Insufficient evidence of randomization
Ávila-Álvarez, A., Alonso-Bidegain, M., De-Rosende-Celeiro, I., Vizcaíno-Cela, M., Larrañeta-Alcalde, L., & Torres-Tobío, G. (2010). Improving social participation of children with autism spectrum disorder: Pilot testing of an early animal-assisted intervention in Spain. *Health & Social Care in the Community, 28*(4), 1220–1229.	Not RCT
Bass, M. M., Duchowny, C. A., & Llabre, M. M. (2009). The effect of therapeutic horseback riding on social functioning in children with autism. *Journal of Autism and Developmental Disorders, 39*(9), 1261–1267.	Included
Becker, J. L., Rogers, E. C., & Burrows, B. (2017). Animal-assisted social skills training for children with autism spectrum disorders. *Anthrozoos, 30*(2), 307–326.	Not RCT
Borgi, M., Loliva, D., Cerino, S., Chiarotti, F., Venerosi, A., Bramini, M., Nonnis, E., Marcelli, M., Vinti, C., De Santis, C., Bisacco, F., Fagerlie, M., Frascarelli, M., & Cirulli, F. (2016). Effectiveness of a standardized equine-assisted therapy program for children with autism spectrum disorder. *Journal of Autism and Developmental Disorders, 46*(1), 1–9.	Included
Burgoyne, L., Dowling, L., Fitzgerald, A., Connolly, M., Browne, J. P., & Perry, I. J. (2014). Parents’ perspectives on the value of assistance dogs for children with autism spectrum disorder: a cross-sectional study. *BMJ Open, 4*(6), Article e004786.	Not RCT
Coman, D. C., Bass, M. P., Alessandri, M., Ghilain, C. S., & Llabre, M. M. (2018). Effect of equine-assisted activities on social and sensory functioning of children with autism. *Society & Animals: Journal of Human-Animal Studies, 26*(6), 551–575.	Included
De Vita, T., Rosa, R., & Napolitano, F. (2018). PET-therapy as an innovative intervention tool in the autism spectrum disorder motor deficits. *Acta Medica Mediterranea, 34*, 1503–1506.	Not RCT
Elmaci, D. T., & Cevizci, S. (2015). Dog-assisted therapies and activities in rehabilitation of children with cerebral palsy and physical and mental disabilities. *International Journal of Environmental Research and Public Health, 12*(5), 5046–5060.	No ASD diagnosis
Funahashi, A., Gruebler, A., Aoki, T., Kadone, H., & Suzuki, K. (2014). Brief Report: The smiles of a child with autism spectrum disorder during an animal-assisted activity may facilitate social positive behaviors-quantitative analysis with smile-detecting interface. *Journal of Autism and Developmental Disorders, 44*(3), 685–693.	Not RCT
Fung, S. C. (2011). *The role of therapy dog in facilitating social interaction for autistic children: An experimental study on animal-assisted play therapy*. Chinese University of Hong Kong.	Insufficient evidence of randomization
Fung, S. C., & Leung, A. S. M. (2014). Pilot study investigating the role of therapy dogs in facilitating social interaction among children with autism. *Journal of Contemporary Psychotherapy, 44*(4), 253–262.	Insufficient evidence of randomization
Gabriels, R. L., Agnew, J. A., Holt, K. D., Shoffner, A., Zhaoxing, P., Ruzzano, S., Clayton, G. H., & Mesibov, G. (2012). Pilot study measuring the effects of therapeutic horseback riding on school-age children and adolescents with autism spectrum disorders. *Research in Autism Spectrum Disorders, 6*(2), 578–588.	No social outcome
Gabriels, R. L., Pan, Z., Dechant, B., Agnew, J. A., Brim, N., & Mesibov, G. (2015). Randomized controlled trial of therapeutic horseback riding in children and adolescents with autism spectrum disorder. *Journal of the American Academy of Child and Adolescent Psychiatry, 54*(7), 541–549.	Included
Gabriels, R. L., Pan, Z., Guérin, N. A., Dechant, B., & Mesibov, G. (2018). Long-term effect of therapeutic horseback riding in youth with autism spectrum disorder: A randomized trial. *Frontiers in Veterinary Science, 5*, Article 156.	Included
Garcia-Gomez, A., Risco, M. L., Rubio, J. C., Guerrero, E., & García-Peña, I. M. (2014). Effects of a program of adapted therapeutic horse-riding in a group of autism spectrum disorder children. *Electronic Journal of Research in Educational Psychology, 12*(1), 107–128.	Insufficient evidence of randomization
Germone, M. M., Gabriels, R. L., Guérin, N. A., Pan, Z., Banks, T., & O’Haire. M. E. (2019). Animal-assisted activity improves social behaviors in psychiatrically hospitalized youth with autism. *Autism, 23*(7), 1740–1751.	Not RCT
Grandgeorge, M., Gautier, Y., Brugaillères, P., Tiercelin, I., Jacq, C., Lebret, M.-C., & Hausberger, M. (2017). Social rivalry triggers visual attention in children with autism spectrum disorders. *Scientific Reports, 7*, Article 10029	No social outcome
Grandgeorge, M., Tordjman, S., Lazartigues, A., Lemonnier, E., Deleau, M., & Hausberger, M. (2012). Does pet arrival trigger prosocial behaviors in individuals with autism? *PLOS ONE, 7*(8), Article e41739	Quasi-experimental design
Grey, A. C. (2008).The effects of therapeutic horseback riding with autistic children. *Dissertation Abstracts International: Section B: The Sciences and Engineering, 68*(11-B), 7663.	Quasi-experimental design
Haight, D. G. (2012). Will the use of therapy dogs provide greater motivation for multiply disabled children. *Dissertation Abstracts International: Section B: The Sciences and Engineering, 72*(8-B), 5009.	Dissertation or conference abstract
Hall, S. S., Wright, H. F., Hames, A., & Mills, D. S. (2016). The long-term benefits of dog ownership in families with children with autism. *Journal of Veterinary Behavior-Clinical Applications and Research, 13*, 46–54.	No social outcome
Hameury, L., Cavagnino, D. T., & Bhat, A. N. (2010). Equine-assisted therapy and autism. *Annales Medico-Psychologiques, 168*(9), 655–659.	Non-English language
Harris, A., & Williams, J. M. (2017). The impact of a horse riding intervention on the social functioning of children with autism spectrum disorder. *International Journal of Environmental Research and Public Health, 14*(7), Article 776.	Insufficient evidence of randomization
Hill, J., Ziviani, J., Driscoll, C., Teoh, A. L., Chua, J. M., & Cawdell-Smith, J. (2020). Canine assisted occupational therapy for children on the autism spectrum: A pilot randomised control trial. *Journal of Autism and Developmental Disorders, 50*(11), 4106–4120.	No social outcome
Holm, M. B., Baird, J. M., Kim, Y. J., Rajora, K. B., D’Silva, D., Podolinsky, L., Mazefsky, C., & Minshew, N. (2014). Therapeutic horseback riding outcomes of parent-identified goals for children with autism spectrum disorder: An ABA’ multiple case design examining dosing and generalization to the home and community. *Journal of Autism and Developmental Disorders, 44*(4), 937–947.	Not RCT
Jenkins, S. R., & Reed, F. D. D. (2013). An experimental analysis of the effects of therapeutic horseback riding on the behavior of children with autism. *Research in Autism Spectrum Disorders, 7*(6), 721–740.	Insufficient evidence of randomization
Katcher, A., & Teumer, S. (2006). A 4 year trial of animal-assisted therapy with public school special education students. In A. Fine (Ed.), *Handbook on animal-assisted therapy: Theoretical foundations and guidelines for practice* (2nd ed., pp. 227–242). Elsevier.	Not RCT
Kern, J. K., Fletcher, C. L., Garver, C. R., Mehta, J. A., Grannemann, B. D., Knox, K. R., Richardson, T. A., & Trivedi, M. H. (2011). Prospective trial of equine-assisted activities in autism spectrum disorder. *Alternative Therapies in Health and Medicine, 17*(3), 14–20.	Not RCT
Kregiel, A., Zaworski, K., & Kolodziej, E. (2019). Effects of animal-assisted therapy on parent-reported behaviour and motor activity of children with autism spectrum disorder. *Health Problems of Civilization, 13*(4), 273–278.	Insufficient evidence of randomization
Kwon, S., Sung, I. Y., Ko, E. J., & Kim, H. S. (2019). Effects of therapeutic horseback riding on cognition and language in children with autism spectrum disorder or intellectual disability: A preliminary study. *Annals of Rehabilitation Medicine-Arm, 43*(3), 279–288.	No social outcome
Lanning, B. A., Matyastik Baier, M. E., Ivey-Hatz, J., Krenek, N., & Tubbs, J. D. (2014). Effects of equine assisted activities on autism spectrum disorder. *Journal of Autism and Developmental Disorders, 44*(8), 1897–1907.	Insufficient evidence of randomization
Morales-Moreno, I., Cerezo-Chuecos, F., Balanza-Galindo, S., Gómez-Díaz, M., & Echevarría-Pérez, P. (2020). Implementation of assisted therapy with dogs in the therapeutic approach to people with autistic spectrum disorder. *Holistic Nursing Practice, 34*(5), 282–290.	Insufficient evidence of randomization
O’Haire, M. E., Mckenzie, S. J., Beck, A. M., & Slaughter, V. (2013). Social behaviors increase in children with autism in the presence of animals compared to toys. *PLOS ONE, 8*(2), Article e57010.	Insufficient evidence of randomization
O’Haire, M. E., McKenzie, S. J., McCune, S., & Slaughter, V. (2013). Effects of animal-assisted activities with guinea pigs in the primary school classroom. *Anthrozoos, 26*(3), 445–458.	Insufficient evidence of randomization
O’Haire, M. E., McKenzie, S. J., McCune, S., & Slaughter, V. (2014). Effects of classroom animal-assisted activities on social functioning in children with autism spectrum disorder. *Journal of Alternative and Complementary Medicine, 20*(3), 162–168.	Insufficient evidence of randomization
Ozyurt, G., Dinsever, Ç., Akpınar, S., Özcan, K., Şal, Y., & Öztürk, Y. (2017). The effect of therapeutic horseback riding for children diagnosed with autism spectrum disorder on autistic symptoms and the quality of life. *Anadolu Psikiyatri Dergisi / Anatolian Journal of Psychiatry, 18*(6), 630–636.	Insufficient evidence of randomization
Ozyurt, G., Ozcan, K., Elikucuk, C. D., Odek, U., & Akpinar, S. (2020). Equine assisted activities have positive effects on children with autism spectrum disorder and family functioning. *Montenegrin Journal of Sports Science and Medicine, 9*(2), 51–58.	Included
Page, C. E. (2013). The social and emotional benefits of therapeutic riding on children with autism spectrum disorder. *Dissertation Abstracts International: Section B: The Sciences and Engineering, 74*(3-B(E)), No-Specified.	Dissertation or conference abstract
Pan, Z., Granger, D. A., Guerin, N. A., Shoffner, A., & Gabriels, R. L. (2019). Replication pilot trial of therapeutic horseback riding and cortisol collection with children on the autism spectrum. *Frontiers in Veterinary Science, 5*, Article 312.	Included
Peters, B. C., Wood, W., Hepburn, S., & Bundy, A. (2020). Pilot Study: Occupational therapy in an equine environment for youth with autism. *OTJR-Occupation Participation and Health, 40*(3), 190–202.	Not RCT
Petrongelli-Halloran, L. M. (2012). Evaluation of prosocial behaviours during animal-assisted therapy for children with pervasive developmental disorders. *Dissertation Abstracts International: Section B: The Sciences and Engineering, 72*(10-B), 6394.	Dissertation or conference abstract
Petty, J. D., Pan, Z., Dechant, B., & Gabriels, R. L. (2017). Therapeutic horseback riding crossover effects of attachment behaviours with family pets in a sample of children with autism spectrum disorder. *International Journal of Environmental Research and Public Health, 14*(3), 256.	No social outcome
Prothmann, A., Albrecht, K., Dietrich, S., Hornfeck, U., Stieber, S., & Ettrich, C. (2005). Analysis of child-dog play behaviour in child psychiatry. *Anthrozoos, 18*(1), 43–58.	No social outcome
Souza-Santos, C., Dos Santos, J. F., Azevedo-Santos, I., & Teixeira-Machado, L. (2018). Dance and equine-assisted therapy in autism spectrum disorder: Crossover randomized clinical trial. *Clinical Neuropsychiatry, 15*(5), 284–290.	Included
Steiner, H., & Kertesz, Z. (2015). Effects of therapeutic horse riding on gait cycle parameters and some aspects of behaviour of children with autism. *Acta Physiologica Hungarica, 102*(3), 324–335.	No social outcome
Steiner, H., & Kertesz, Z. (2012, December 2–5). *Effect of therapeutic riding on Gait Cycle parameters and behavioural skills of autistic children (A controlled study)* [Conference session]. 3rd IEEE International Conference on Cognitive Infocommunications, Kosice.	Not RCT
Uccheddu, S., Albertini, M., Pierantoni, L., Fantino, S., & Pirrone, F. (2019). The impacts of a reading-to-dog programme on attending and reading of nine children with autism spectrum disorders. *Animals, 9*(8), Article 491.	Included
Ward, S. C., Whalon, K., Rusnak, K., Wendell, K., & Paschall, N. (2013). The association between therapeutic horseback riding and the social communication and sensory reactions of children with autism. *Journal of Autism and Developmental Disorders, 43*(9), 2190–2198.	Not RCT
Welsh, K. C. (2010). The use of dogs to impact joint attention in children with autism spectrum disorders. *Dissertation Abstracts International: Section B: The Sciences and Engineering, 70*(7-B), 4500.	Not RCT
Wild, D. L. (2013). The impact of canine assistance for children with autism and the family unit. *Dissertation Abstracts International Section A: Humanities and Social Sciences, 73*(8-A(E)), No-Specified.	Dissertation or conference abstract
Wright, H., Hall, S., Hames, A., Hardiman, J., Mills, R., PAWS Team, & Mills, D. S. (2015). Acquiring a pet dog significantly reduces stress of primary carers for children with autism spectrum disorder: A prospective case control study. *Journal of Autism & Developmental Disorders, 45*(8), 2531–2540.	No social outcome
Wright, H., Hall, S., Hames, A., Hardiman, J., Mills, R., PAWS Team, & Mills, D. S. (2015). Pet dogs improve family functioning and reduce anxiety in children with autism spectrum disorder. *Anthrozoos, 28*(4), 611–624.	No social outcome

10 Full texts. Search – 28 October 2020 – 8 October 2021.

**Table:**

Reference	Reason for exclusion
Ben-Itzchak, E., & Zachor, D. A. (2021). Dog training intervention improves adaptive social communication skills in young children with autism spectrum disorder: A controlled crossover study. *Autism, 25*(6), 1682–1693. https://doi.org/10.1177/13623613211000501	Insufficient evidence of randomization
Carlisle, G. K., Johnson, R. A., Wang, Z., Bibbo, J., Cheak-Zamora, N., & Lyons, L. A. (2021). Exploratory study of cat adoption in families of children with autism: Impact on children’s social skills and anxiety. *Journal of pediatric nursing, 58*, 28–35. https://doi.org/10.1016/j.pedn.2020.11.011	Not an AAI
Doney, E. (2021). Animal-assisted interventions with dogs: A review of the current literature [Health & Mental Health Treatment & Prevention 3300]. *Dissertation Abstracts International: Section B: The Sciences and Engineering, 82*(6-B), No-Specified. http://ovidsp.ovid.com/ovidweb.cgi?T = JS&PAGE = reference&D = psyc17&NEWS = N&AN = 2021-08068-014	A review
Hernandez-Espeso, N., Martinez, E. R., Sevilla, D. G., & Mas, L. A. (2021). Effects of dolphin-assisted therapy on the social and communication skills of children with autism spectrum disorder. *Anthrozoos, 34*(2), 251–266. https://doi.org/10.1080/08927936.2021.1885140	Included
Kemeny, B., Hutchins, D., Burk, S., & Gramlich, C. (2021). Therapeutic riding or mindfulness: Comparative effectiveness of two recreational therapy interventions for adolescents with autism. *Journal of Autism and Developmental Disorders*. Advance online publication. https://doi.org/10.1007/s10803-021-05136-z	Not children with ASD
Lobato Rincon, L. L., Rivera Martin, B., Medina Sanchez, M. A., Villafaina, S., Merellano-Navarro, E., & Collado-Mateo, D. (2021). Effects of dog-assisted education on physical and communicative skills in children with severe and multiple disabilities: A pilot study. *Animals, 11*(6), 1741. https://doi.org/10.3390/ani11061741	Not RCT
Peters, B. C., Wood, W., Hepburn, S., & Merritt, T. (2021). The feasibility and acceptability of occupational therapy in an equine environment for youth with autism spectrum disorder. *Research in Autism Spectrum Disorders, 80*, Article 101695. https://doi.org/10.1016/j.rasd.2020.101695	Not RCT
Peters, B. C., Wood, W., Hepburn, S., & Moody, E. J. (2021). Preliminary efficacy of occupational therapy in an equine environment for youth with autism spectrum disorder. *Journal of Autism and Developmental Disorders*. Advance online publication. https://doi.org/10.1007/s10803-021-05278-0	Included
Wijker, C., Leontjevas, R., Enders-Slegers, M.-J., Kupper, N., & Spek, A. (2021). The effects of animal assisted therapy on autonomic and endocrine activity in adults with autism spectrum disorder: A randomized controlled trial. *General Hospital Psychiatry, 72*, 36–44. https://doi.org/10.1016/j.genhosppsych.2021.05.003	No social outcome
Zhao, M., Chen, S., You, Y., Wang, Y., & Zhang, Y. (2021). Effects of a therapeutic horseback riding programme on social interaction and communication in children with autism. *International Journal of Environmental Research and Public Health, 18*(5), Article 2656. https://doi.org/10.3390/ijerph18052656	Included

RCT: randomized controlled trial; AAI: animal-assisted intervention; ASD: autism spectrum disorder.
